# Entropy and Gravitation—From Black Hole Computers to Dark Energy and Dark Matter

**DOI:** 10.3390/e21111035

**Published:** 2019-10-25

**Authors:** Y. Jack Ng

**Affiliations:** Institute of Field Physics, Department of Physics and Astronomy, University of North Carolina, Chapel Hill, NC 27599, USA; yjng@physics.unc.edu

**Keywords:** entropy, gravitation, spacetime foam, quantum foam, holography, dark energy, dark matter, infinite statistics, turbulence

## Abstract

We show that the concept of entropy and the dynamics of gravitation provide the linchpin in a unified scheme to understand the physics of black hole computers, spacetime foam, dark energy, dark matter and the phenomenon of turbulence. We use three different methods to estimate the foaminess of spacetime, which, in turn, provides a back-door way to derive the Bekenstein-Hawking formula for black hole entropy and the holographic principle. Generalizing the discussion for a static spacetime region to the cosmos, we find a component of dark energy (resembling an effective positive cosmological constant of the correct magnitude) in the current epoch of the universe. The conjunction of entropy and gravitation is shown to give rise to a phenomenological model of dark matter, revealing the natural emergence, in galactic and cluster dynamics, of a critical acceleration parameter related to the cosmological constant; the resulting mass profiles are consistent with observations. Unlike ordinary matter, the quanta of the dark sector are shown to obey infinite statistics. This property of dark matter may lead to some non-particle phenomenology and may explain why dark matter particles have not been detected in dark matter search experiments. We also show that there are deep similarities between the problem of “quantum gravity” (more specifically, the holographic spacetime foam) and turbulence.

## 1. Introduction

What is the difference between a computer and a black hole? This question is not a joke, but is an intriguing problem in modern physics [1]. The reason can be traced to the fact that all physical systems are computers. Every elementary particle stores bits of data, and every time two such particles interact, those bits are transformed. Black holes are merely the most exotic example of the general principle that the universe registers and processes information.

The principle is not new. In the 19th century, the founders of statistical mechanics developed what would later be called information theory to explain the laws of thermodynamics. The key player in information theory is entropy *S*, the macroscopic thermodynamic quantity characterizing disorder. The second law of thermodynamics stipulates that disorder as embodied by entropy always increases. Entropy *S* can be written in terms of a microscopic probabilistic quantity *W* as S=klogW, where *k* is the Boltzmann constant. Deeply ingrained in probabilities, *S* finds its true home in quantum mechanics. And the confluence of physics and information theory flows from the central maxim of quantum mechanics: at bottom, nature is discrete. It is the quantum-mechanical nature of information that is responsible for the computational ability of black holes; without quantum effects, a black hole would destroy, rather than process, information.

Black holes, though exotic, are, in a way, the simplest gravitational systems. To examine the properties of black hole computers, we can start with a more general discussion of aspects of quantum gravity, the synthesis of quantum mechanics and general relativity. If space-time, like every thing else, undergoes quantum mechanical fluctuations, then space is composed of an ever-changing arrangement of bubbles which John Wheeler called spacetime foam, also known as quantum foam [2,3,4]. As we will show, quantum fluctuations of spacetime determine the precision with which the geometry of spacetime can be measured and they limit the power of computers in general, black hole computers in particular. Applied to cosmology, spacetime foam physics leads to the prediction of a dark energy component in the current Universe (of the correct magnitude). Combined with ideas from gravitational thermodynamics and entropic gravity, we are led to a phenomenological model of dark matter in which a critical acceleration parameter, related to the (effective) cosmological constant, emerges.

This review article on entropy and gravitation is organized as follows: In Section 2.1, we discuss a gedanken experiment to measure the foaminess of spacetime, more specifically the induced uncertainties in distance (and time) measurements. In Section 2.2 we rederive these results by the method of mapping the geometry of spacetime, which also provides a way to derive the holographic principle. The results for spacetime fluctuations are then applied to the discussion of black holes in Section 3 to deduce black-hole entropy, lifetime and power as a computer. The discussion for a static spacetime region with low spatial curvature in Section 2 is generalized, in Section 4, to the case of an expanding universe, uncovering the constituents of dark energy (of the correct magnitude in the present era of the universe) in the form of (extremely) long-wavelength quanta which, thus, act like a positive cosmological constant. In this section we also argue how the results found for spacetime fluctuations indicate why the universe necessarily contains more than ordinary matter. (One may even suggest that quantum gravity, in combination with thermodynamics, naturally demands the existence of a dark sector.) Section 4.2 is used to show that the quanta of dark energy, unlike ordinary matter, obey an exotic statistics known as infinite statistics (also known as quantum Boltzmann statistics). Another method to infer (and to check the consistency of the results for) spacetime fluctuations and the magnitude of dark energy is given in Section 6 by applying causal set theory and unimodular gravity. Section 7 is devoted to the construction of a phenomenological dark matter model (called Modified Dark Matter (MDM)) by generalizing gravitational thermodynamics and entropic gravity arguments to a spacetime with positive cosmological constant (like ours). Then we show that dark matter quanta (like dark energy) obey infinite statistics and briefly enumerate some of MDM’s quantitative and qualitative successes (so far). In Section 8, we show some deep similarities between the physics of spacetime foam and turbulence. We give a short conclusion in Section 8. There are two appendices: In Appendix A, we discuss energy-momentum fluctuations and some possible tests of spacetime foam. For completeness we give a short introduction to the subject of infinite statistics in Appendix B.

On notations, the subscript “P” denotes Planck units; thus $l_{P}\equiv {\left(\hbar G/c^{3}\right)}^{1/2}∼10^{-33}$ cm is the Planck length and so forth. On units, $k_{B}$ (the Boltzmann constant) and *ℏ* and c are often put equal to 1 for simplicity.

## 2. Quantum Fluctuations of Spacetime

At small scales, spacetime is fuzzy and foamy due to quantum fluctuations. One manifestation of the fluctuations is in the induced uncertainties in any distance measurement. We will derive the uncertainties or fluctuations by two independent methods [1,5,6] in the following two subsections.

### 2.1. Gedanken Experiment

Consider the following experiment to measure the distance *l* between two points. Following Wigner [7], we put a clock at one of the points and a mirror at the other. By sending a light signal from the clock to the mirror in a timing experiment, we can determine the distance. However, the quantum uncertainty in the positions of the clock and the mirror introduces an inaccuracy δl in the distance measurement. Let us concentrate on the clock (of mass *m*). If it has a linear spread δl when the light signal leaves the clock, then its position spread grows to $\delta l+\hbar l{\left(mc\delta l\right)}^{-1}$ when the light signal returns to the clock, with the minimum at $\delta l={\left(\hbar l/mc\right)}^{1/2}$. Hence one concludes that

(1) $$\delta l^{2}≳\frac{\hbar l}{mc}.$$

One can supplement this requirement with a limit from general relativity [5,6]. To wit, let the clock be a light-clock consisting of two mirrors (each of mass m/2), a distance *d* apart, between which bounces a beam of light. For the uncertainty in distance measurement not to be greater than δl, the clock must tick off time fast enough so that d/c≲δl/c. But *d*, the size of the clock, must be larger than the Schwarzschild radius $Gm/c^{2}$ of the clock, for otherwise one cannot read the registered time. From these two conditions, it follows that

(2) $$\delta l≳\frac{Gm}{c^{2}},$$

the product of which with Equation (1) yields [5,6,8,9]

(3) $$\delta l≳{\left(ll_{P}^{2}\right)}^{1/3}=l_{P}{\left(\frac{l}{l_{P}}\right)}^{1/3}.$$

A gedanken experiment to measure a time interval *T* gives an analogous expression: $\delta T≳{\left(Tt_{P}^{2}\right)}^{1/3}.$

### 2.2. Mapping the Geometry of Spacetime

Since quantum fluctuations of spacetime manifest themselves in the form of uncertainties in the geometry of spacetime, the structure of spacetime foam can be inferred from the accuracy with which we can measure that geometry [1]. Let us consider a spherical volume of radius *l* over the amount of time T=2l/c it takes light to cross the volume. One way to map out the geometry of this spacetime region is to fill the space with clocks, exchanging signals with other clocks and measuring the signals’ times of arrival. This process of mapping the geometry is a sort of computation, in which distances are gauged by transmitting and processing information; hence the total number of operations is bounded by the Margolus-Levitin theorem [10] in quantum computation, which stipulates that the rate of operations for any computer cannot exceed the amount of energy *E* that is available for computation divided by πℏ/2. A total mass *M* of clocks then yields, via the Margolus-Levitin theorem, the bound on the total number of operations given by $(2Mc^{2}/\pi \hbar )\times 2l/c$. But to prevent black hole formation, *M* must be less than $lc^{2}/2G$. Together, these two limits imply that the total number of operations that can occur in a spatial volume of radius *l* for a time period 2l/c is no greater than $∼{\left(l/l_{P}\right)}^{2}$. (Here and henceforth we neglect multiplicative constants of order unity and set c=1=ℏ.) To maximize spatial resolution, each clock must tick only once during the entire time period. And if we regard the operations partitioning the spacetime volume into “cells”, then on the average each cell occupies a spatial volume no less than $∼l^{3}/\left(l^{2}/l_{P}^{2}\right)=ll_{P}^{2}$, yielding an average separation between neighboring cells no less than $l^{1/3}l_{P}^{2/3}$. This spatial separation is interpreted as the average minimum uncertainty in the measurement of a distance *l*, that is, $\delta l≳l^{1/3}l_{P}^{2/3}$, in agreement with the result obtained in the previous subsection. (We will use yet another argument to check this result in Section 5).

As an application, we can now heuristically derive the holographic principle. Since, on the average, each cell occupies a spatial volume of $ll_{P}^{2}$, a spatial region of size *l* can contain no more than $l^{3}/\left(ll_{P}^{2}\right)={\left(l/l_{P}\right)}^{2}$ cells. Thus this spacetime foam model corresponds to the case of maximum number of bits of information $l^{2}/l_{P}^{2}$ in a spatial region of size *l*, that is allowed by the holographic principle [11,12,13,14], according to which, the maximum amount of information stored in a region of space scales as the area of its two-dimensional surface, like a hologram. Accordingly, we will refer to this spacetime foam model (corresponding to $\delta l≳l^{1/3}l_{P}^{2/3}$) as the holographic spacetime foam model.

## 3. Clocks, Computers and Black Holes

In this section we will show that the properties of black holes are inextricably intertwined with those of spacetime. For example, the strange scaling of space fluctuations with the cube root of distances provide a back-door way to derive the Bekenstein-Hawking formula for black hole memory [15,16].

But let us first consider a clock (technically, a simple and “elementary” clock, not composed of smaller clocks that can be used to read time separately or sequentially, with a black hole clock being the limiting example), capable of resolving time to an accuracy of *t*, for a period of *T* (the running time or lifetime of the clock). Then bounds on the resolution time and the lifetime of the clock can be derived by following an argument very similar to that used above in the analysis of the gedanken experiment to measure distances. Actually, the two arguments are so similar that one can identify the corresponding quantities:

(4) δl/c↔t;l/c↔T.

It follows that the following limits [7,17,18] hold:

(5) $$t^{2}≳\frac{\hbar T}{mc^{2}},\;t≳\frac{Gm}{c^{3}},\;T/t^{3}≲t_{P}^{-2}=\frac{c^{5}}{\hbar G},$$

which are, respectively, the analogues of Equations (1)–(3).

One can easily translate the relations for clocks given above into useful relations for a simple computer (technically, it refers to a computer designed to perform highly serial computations, that is, one that is not divided into subsystems computing in parallel—like a black hole computer which acts as a single unit). Let ν denote the clock rate of the computer, that is, the number of operations per bit per unit time and *I* the number of bits of information in the memory space of a simple computer. Then one can identify the corresponding quantities for simple clocks and simple computers as

(6) $$\frac{1}{t}↔\nu ;\;\frac{T}{t}↔I.$$

Now we can apply what we have learned about clocks and computers to black holes [17,18]. Let us consider using a black hole to measure time. It is reasonable to use the light travel time around the black hole’s horizon as the resolution time of the clock, that is, $t∼\frac{Gm}{c^{3}}\equiv t_{BH}$, then from the last of Equation (5), one immediately finds that $T∼\frac{G^{2}m^{3}}{\hbar c^{4}}\equiv T_{BH}$, recovering Hawking’s result for the black hole’s lifetime. (Note that the lifetime bound is saturated for black holes.)

Applying the results for $T_{BH}$ and $t_{BH}$, we readily find the number of bits in the memory space of a black hole computer as $I=\frac{T_{BH}}{t_{BH}}∼\frac{m^{2}}{m_{P}^{2}}∼\frac{r_{S}^{2}}{l_{P}^{2}}$, where $m_{P}=\hbar /\left(t_{P}c^{2}\right)$ is the Planck mass, *m* and $r_{S}^{2}$ denote the mass and event horizon area of the black hole respectively. This gives the number of bits *I* as the event horizon area in Planck units, in agreement with the identification of black hole entropy [15,16]. (Recall that entropy *S* and the number of bits *I* are related by $S=k_{B}I\ln2$.)

All these results reinforce the conceptual interconnections of the physics underlying spacetime foam, black holes and computation. It is interesting that all black hole computers obey the universal relation (obtained by using the computer analogue of the last equation in Equation (5)): $I\nu^{2}∼c^{5}/\hbar G$, which mathematically demonstrates the linkage between information and the theories of special relativity (where the defining parameter is c), general relativity (G) and quantum mechanics (*ℏ*).

## 4. Dark Energy

We can now apply the insights we have learned from the fine-scale structure of spacetime to cosmology and fundamental physics to learn the behavior of cosmic dark energy.

### 4.1. Spacetime Foam and Dark Energy

As shown in Section 2.2 on mapping the geometry of space-time, maximum spatial resolution (which leads to the holographic bound) requires maximum energy density (that is allowed to avoid the collapse into a black hole) given by

(7) $$\rho ∼\frac{l/G}{l^{3}}={\left(ll_{P}\right)}^{-2}.$$

Let us now generalize this discussion for a static spacetime region with low spatial curvature to the case of an expanding universe by substituting *l* by 1/H, where *H* is the Hubble parameter [19,20,21]. Equation (7) yields the cosmic energy density $\rho ∼{\left(\frac{H}{l_{P}}\right)}^{2}∼{\left(R_{H}l_{P}\right)}^{-2}$. Next, recall that we have also shown that the Universe contains $I∼{\left(R_{H}/l_{P}\right)}^{2}$ bits of information ($∼10^{122}$ for the current epoch) [19,20]. Hence the average energy carried by each of these bits or quanta is $\rho R_{H}^{3}/I∼R_{H}^{-1}$. These long-wavelength bits or “particles” carry negligible kinetic energy. (Note the quotations around the word “particles”. Such long-wavelength quanta can hardly be called particles. We will simply call them “particles”.) Since pressure (energy density) is given by kinetic energy minus (plus) potential energy, a negligible kinetic energy means that the pressure of the unconventional energy is roughly equal to minus its energy density, leading to accelerating cosmic expansion, in agreement with observation [22,23]. This scenario is very similar to that of quintessence, but it has its origin in local small scale physics—specifically, the holographic spacetime foam [24,25,26]. Thus intriguingly, the large-scale ($∼R_{H}$) physics of dark energy is intimately connected to the small-scale ($∼R_{H}^{1/3}l_{P}^{2/3}$) physics of spacetime foam.

Alternatively one can interpret these long-wavelength quanta as constituents of dark energy, contributing a more or less uniformly distributed cosmic energy density and hence acting as a dynamical effective cosmological constant

(8) $$\Lambda ∼H^{2},$$

a result for the magnitude of Λ that will be checked in the next section.

As a corollary to the above discussion, we can now give a heuristic argument [1,19,20,21] (based on quantum gravity consideration) on why the Universe cannot contain ordinary matter only. Start by assuming the Universe (of size $l=R_{H}$) has only ordinary matter and hence all information is stored in ordinary matter. According to the statistical mechanics for ordinary matter at temperature *T*, energy scales as $E∼l^{3}T^{4}$ and entropy goes as $S∼l^{3}T^{3}$. Black hole physics can be invoked to require $E≲\frac{l}{G}=\frac{l}{l_{P}^{2}}$. Then it follows that the entropy *S* and hence also the number of bits *I* (or the number of degrees of freedom on ordinary matter) are bounded by $≲{\left(l/l_{P}\right)}^{3/2}$. We can repeat verbatim the argument given in Section 2 on the relationship between the bound on the number of degrees of freedom in a region with volume $l^{3}$ and δl, the quantum fluctuation of distance *l*, to conclude that, if only ordinary matter exists, $\delta l≳{\left(\frac{l^{3}}{{\left(l/l_{P}\right)}^{3/2}}\right)}^{1/3}=l^{1/2}l_{P}^{1/2}$ which is much greater than $l^{1/3}l_{P}^{2/3}$, the result found above from our analysis of the Salecker-Wigner type of gedanken experiments and implied by the holographic principle. It is now apparent that ordinary matter contains only an amount of information dense enough to map out spacetime at a level with much coarser spatial resolution. Thus, there must be other kinds of matter/energy with which the Universe can map out its spacetime geometry to a finer spatial accuracy than is possible with the use of conventional ordinary matter. We conclude that a dark sector indeed exists in the Universe! One can draw this conclusion, independent of recent observations of dark energy and dark matter. We also note that the ($∼{\left(R_{H}/l_{P}\right)}^{2}$) bits/“particles” of dark energy vastly outnumber the ($∼{\left(R_{H}/l_{P}\right)}^{3/2}$) particles of ordinary matter by an enormously huge factor of ${\left(R_{H}/l_{P}\right)}^{1/2}∼10^{31}$ for the present observable universe.

### 4.2. Dark Energy as Quanta of Infinite Statistics

According to the holographic spacetime foam model, the constituents of dark energy are quanta/“particles” with very long wavelengths (of the order of Hubble radius $R_{H}$). Consider $N∼{\left(R_{H}/l_{P}\right)}^{2}$ such “particles” and let us assume that they obey Boltzmann statistics in volume $V∼R_{H}^{3}$ at $T∼R_{H}^{-1}$, the average energy carried by each “particle”. The partition function $Z_{N}={\left(N!\right)}^{-1}{\left(V/\lambda^{3}\right)}^{N}$ gives the entropy of the system $S=N[ln\left(V/N\lambda^{3}\right)+5/2]$, with thermal wavelength $\lambda ∼T^{-1}∼R_{H}$. But then $V∼\lambda^{3}$, so *S* becomes negative unless N∼1 which is equally nonsensical. A simple solution is to stipulate that the *N* inside the log in *S*, i.e, the Gibbs factor ${\left(N!\right)}^{-1}$ in $Z_{N}$, must be absent. (This means that the N “particles” are distinguishable!) Then the entropy is positive: $S=N[ln\left(V/\lambda^{3}\right)+3/2]∼N$. Now, the only known consistent statistics in greater than 2 space dimensions without the Gibbs factor is the quantum Boltzmann statistics, also known as infinite statistics [27,28,29] (See Appendix B for a succinct description of this exotic statistics). Thus we conclude (at least are led to speculate) that the “particles” constituting dark energy obey infinite statistics, rather than the familiar Fermi or Bose statistics [21,30]. This is the over-riding difference between dark energy and conventional matter. Note that here it is the physical non-negativity requirement of entropy for a gravitational system that leads to this unexpected conclusion.

## 5. From Causal-Set Theory and Unimodular Gravity to Space-Time Foam

In this section we will rederive the magnitudes of δl (Equation (3)) and Λ (Equation (8)) by using causal-set theory and (generalized) unimodular gravity. The causal-set theory [31] stipulates that continuous geometries in classical gravity should be replaced by “causal-sets”, the discrete substratum of spacetime. In the framework of the causal-set theory, the fluctuation in the number of elements *N* making up the set is of the Poisson type, that is, $\delta N∼\sqrt{N}$. For a causal set, the spacetime volume $V_{st}$ becomes $l_{P}^{4}N$. It follows that

(9) $$\delta V_{st}∼G\sqrt{V_{st}}.$$

As in Section 2.2, let us consider a spherical volume of radius *l* over the amount of time T=2l/c it takes light to cross the volume. We want to find the minimum of δl; so $\delta V_{st}∼T{\left(\delta l\right)}^{3}∼l{\left(\delta l\right)}^{3}$. With the help of Equation (9) and $\sqrt{V_{st}}∼l^{2}$, we recover $\delta l≳{\left(ll_{P}^{2}\right)}^{1/3}$.

As a check on Equation (8), we will make use of the theory of unimodular gravity [32,33,34,35,36,37,38], more specifically its generalized action given by the Henneaux and Teitelboim action $S_{unimod}=-{\left(16\pi G\right)}^{-1}\int \left[\sqrt{g}\left(R+2\Lambda \right)-2\Lambda \partial_{\mu }T^{\mu }\right]\left(d^{3}x\right)dt.$ In this theory, Λ/G plays the role of “momentum” conjugate to the “coordinate” $\int d^{3}xT_{0}$ which can be identified as the spacetime volume $V_{st}$. Hence the fluctuations of Λ/G and $V_{st}$ obey a quantum uncertainty principle, $\delta V_{st}\;\delta \Lambda /G∼1.$ This, together with Equation (9), yields $\delta \Lambda ∼V_{st}^{-1/2}∼R_{H}^{-2}∼H^{2}$, where we have used $∼R_{H}^{4}$ for the whole spacetime volume $V_{st}$ with $R_{H}$ being the Hubble radius. Finally, following Baum [39] and Hawking [40], we can argue [34,35,36,37] that, in the framework of unimodular gravity, Λ vanishes to the lowest order of approximation (i.e., Λ=0 dominates the path integral of the Euclidean vacuum functional) and that its first order correction is positive (at least for the the cosmic epoch corresponding to redshift $z\overset{<}{∼}1$.) We conclude that $\Lambda ∼+H^{2}$, contributing a cosmic energy density ρ given by $\rho \;∼\;\frac{1}{l_{P}^{2}R_{H}^{2}},$ as observed.

## 6. Dark Matter

The standard cosmological model, ΛCDM, has been very successful. But aside from the fact that dark matter particles have not been (directly) detected, this model suffers some noticeable shortcomings, such as missing satellite problem, core/cusp problem, too-big-to-fail problem, to name just a few [41]. There are also two serious problems that CDM proponents have to face: CDM theories fail to explain in a natural way [42] the baryonic Tully-Fisher relation (the asymptotic velocity-mass $v^{4}\propto M$ relation) [43] for galaxies and the presence of a universal acceleration scale in galactic (and cluster) dynamics [44,45]. These apparent shortcomings of ΛCDM motivated the author and his collaborators to construct the Modified Dark Matter (MDM) [46,47,48], a phenomenological dark matter model inspired by the consideration of entropy and gravitation. Our approach can be traced to the work of Jacobson on gravitational thermodynamics [49] and the work of Verlinde on entropic gravity [50,51,52].

### 6.1. From Gravitational Thermodynamics /Entropic Gravity to MDM

Entropy and gravitation come together in Jacobson’s idea of gravitational thermodynamics. Essentially Jacobson proposes that gravity is simply a consequence of disorder as quantified by entropy. Applying Bekenstein’s idea of black hole entropy [15] and Unruh’s formula [53,54] for the temperature experienced by an accelerating body, Jacobson is able to derive Einstein’s equation. His work is instrumental in inspiring Verlinde’s formulation of entropic gravity which is appropriately generalized in the construction of Modified Dark Matter.

In order to appreciate how important a role entropy and gravitation play, let us first summarize the crucial steps in Verlinde’s derivation of the canonical Newton’s laws.

(I) Newton’s 2nd law $\vec{F}=m\vec{a}$:
  (a) Verlinde uses the first law of thermodynamics to propose the concept of entropic force $F_{entropic}=T\frac{\Delta S}{\Delta x}.$
  (b) Then he invokes Bekenstein’s original arguments concerning the entropy *S* of black holes: $\Delta S=2\pi k_{B}\frac{mc}{\hbar }\Delta x$.
  (c) Finally he applies the formula for the Unruh temperature, $k_{B}T=\frac{\hbar a}{2\pi c},$ associated with a uniformly accelerating (Rindler) observer.

(II) Newton’s law of gravity $a=GM/r^{2}$:
  (a) Verlinde considers an imaginary quasi-local (spherical) holographic screen of area $A=4\pi r^{2}$ with temperature *T*.
  (b) Then he uses equipartition of energy $E=\frac{1}{2}Nk_{B}T$ with $N=Ac^{3}/\left(G\hbar \right)$ being the total number of degrees of freedom (bits) on the screen.
  (c) Finally he applies the Unruh temperature formula and $E=Mc^{2}$.

We can now construct MDM by generalizing Verlinde’s proposal to de Sitter (dS) space with positive cosmological constant Λ (like our accelerating universe). In such a dS space, the Unruh-Hawking temperature, as measured by an inertial observer, is $T_{dS}=\frac{1}{2\pi k_{B}}a_{0}$ where $a_{0}=\sqrt{\Lambda /3}∼H$. The net temperature as measured by the non-inertial observer [55,56] (due to some matter sources that cause the acceleration *a*) is $\tilde{T}\equiv T_{dS+a}-T_{dS}=\frac{1}{2\pi k_{B}}\left[\sqrt{a^{2}+a_{0}^{2}}-a_{0}\right]$.

Part (I) of Verlinde’s argument can now be generalized to yield the entropic force (in de Sitter space) $F_{entropic}=\tilde{T}\;\nabla_{x}S=m\left[\sqrt{a^{2}+a_{0}^{2}}-a_{0}\right]$. For $a\gg a_{0}$, we have $F_{entropic}\approx ma$. For the small acceleration $a\ll a_{0}$ regime (where the galactic rotation curves are observed to be flat and the Tully-Fisher relation holds): $F_{entropic}\approx m\frac{a^{2}}{2\;a_{0}},$ which, after some algebra, can be shown to be equal to $F_{Milgrom}\approx m\sqrt{a_{N}a_{c}}\;$ the force law proposed by Milgrom in his theory of modified Newtonian dynamics (MOND) [42] at the galactic scale. Here we have identified $a_{0}\approx 2\pi a_{c}$, with the critical galactic acceleration $a_{c}∼\sqrt{\Lambda /3}∼H∼10^{-8}cm/s^{2}$. Thus we have correctly predicted the magnitude of $a_{c}$ (which Milgrom puts in by hand). From our perspective, MOND is a phenomenological consequence of quantum gravity. But while MOND is successful in describing galactic dynamics, it is considerably less so at the cluster and cosmic scales.

Part (II) of Verlinde’s argument is straightforwardly generalized to give $2\pi k_{B}\tilde{T}=\frac{G\;\tilde{M}}{r^{2}}$, where $\tilde{M}=M+M^{\prime }$ represents the total mass enclosed within the volume $V=4\pi r^{3}/3$, with $M^{\prime }$ being some unknown mass, that is, dark matter. It can be checked that consistency demands $M^{\prime }=\frac{1}{\pi }\;{\left(\;\frac{a_{0}}{a}\;\right)}^{2}\;M$. It is noteworthy that dark matter ($M^{\prime }$) is related to dark energy (codified in $a_{0}$) and baryonic matter (M) in MDM. Succinctly the force law in MDM is given by $F_{entropic}=m\left[\sqrt{a^{2}+a_{0}^{2}}-a_{0}\right]=m\;a_{N}\left[\;1+{\left(a_{0}/a\right)}^{2}/\pi \right]$. Recall that, in the small acceleration $a\ll a_{0}$ regime, MDM behaves like MOND. Thus dark matter ($M^{\prime }$) of the kind we have in MDM can behave as if there is no (dark) matter but MOND (which denies the existence of dark matter); for this reason, initially [46,47,48,57] we called our dark matter model “MONDian Dark Matter” with which Modified Dark Matter shares the acronym MDM.

### 6.2. Quanta of MDM Obey Infinite Statistics

It has been known [58,59] that the MONDian force law can be formulated as being governed by a nonlinear generalization of Poisson’s equation which describes the nonlinear electrostatics embodied in the Born-Infeld theory. It is therefore useful to reformulate MDM, via an effective gravitational dielectric medium, motivated by the analogy between Coulomb’s law in a dielectric medium and Milgrom’s law for MOND. Starting from the Born-Infeld theory of electrostatics, we can write the corresponding gravitational Hamiltonian density in the form $H_{g}=\left(\;\sqrt{A^{2}+A_{0}^{2}}-A_{0}\;\right)/\left(4\pi \right)$ in terms of the local gravitational fields $\vec{A}$ and ${\vec{A}}_{0}$. As in the Verlinde approach, let us assume energy equipartition. Then the effective gravitational Hamiltonian density is equal to $H_{g}=\frac{1}{2}\;k_{B}\;T_{\mathrm{eff}}\;$. The Unruh temperature formula $T_{\mathrm{eff}}=\frac{\hbar }{2\;\pi \;k_{B}}\;a_{\mathrm{eff}}\;$ implies that the effective acceleration is given by $a_{\mathrm{eff}}=\sqrt{A^{2}+A_{0}^{2}}-A_{0}\;$, which becomes $a_{\mathrm{eff}}=\sqrt{a^{2}+a_{0}^{2}}-a_{0}\;$ upon the identification (with the help of the equivalence principle) of the local accelerations $\vec{a}$ and ${\vec{a}}_{0}$ with the local gravitational fields $\vec{A}$ and ${\vec{A}}_{0}$ respectively. Thus the Born-Infeld inspired force law takes the form of the MDM force law!

Next recall that the equipartition theorem in general states that the average of the Hamiltonian is given by $\langle H\rangle =-\frac{\partial \log Z(\beta )}{\partial \beta }\;$, where $\beta^{-1}=k_{B}T$ and *Z* denotes the partition function. To obtain $\langle H\rangle =\frac{1}{2}\;k_{B}\;T$ per degree of freedom, even for very low temperature, we require *Z* to be of the Boltzmann form Z=exp(−βH). But this is precisely what is called the infinite statistics. (See Appendix B.) Thus we have shown that the quanta of MDM (like those of dark energy as shown in Section 4) obey infinite statistics [57].

### 6.3. Observational Tests of MDM

*Tests at both the galactic and cluster scales* [60,61,62,63].

Since such tests have been described in a long review article [63], here we do not have to go into details. Let us just recall that we have found the emergence of a critical acceleration parameter related to Λ in MDM and it is found in correlations between dark matter and baryonic matter in galaxy rotation curves. The resulting MDM mass profiles are consistent with observational data at both the galactic and cluster scales. (We can point out that MDM is more economical than CDM in fitting data at the galactic scale and it is superior to MOND at the cluster scale.) Logically (and happily as it indeed turns out to be the case), the same critical acceleration appears both in the galactic and cluster data fits based on MDM.

There is one technical point that is worth mentioning. It is related to the fact that galaxies and clusters have very different length scales. Recall that, in our construction of MDM, we re-interpret acceleration in terms of temperature of the Unruh-Hawking kind. Thus, in principle, the mass profile $M^{\prime }=\frac{1}{\pi }\;{\left(\;\frac{a_{0}}{a}\;\right)}^{2}\;M$, fixed by the ratio of the corresponding Unruh-Hawking temperatures, can be altered due to some physical effects associated with a change of scale. For example, in the presence of gravity, the temperature is not constant in space at equilibrium. As a result, it can be modified due to the Tolman-Ehrenfest effect [64,65]. Such an effect must be incorporated in working out successfully the dark matter density profiles [61,62,63].

MDM and Cosmology

To apply MDM to cosmology, we must replace $\tilde{M}$ (a non-relativistic source) with the active gravitational (Tolman-Komar) mass (a fully relativistic source). In that case, we have $\sqrt{a^{2}+a_{0}^{2}}-a_{0}=\frac{G\;(\;M\left(t\right)+M^{\prime }\left(t\right)\;)}{{\tilde{r}}^{2}}+4\pi G\;p\;\tilde{r}-\frac{\Lambda }{3}\;\tilde{r}$, where *p* stands for pressure and $\tilde{r}$ is the physical radius. Then it can be shown [46] that the Friedmann equations are recovered. Note that if we naively use MOND at the cluster or cosmic scale, we would be missing the pressure and cosmological constant terms, which could be significant. This may explain why MOND does not work well at the cluster and cosmic scales, whereas MDM works at both the galactic and cluster scales and is expected to be completely compatible with cosmology [46].

MDM and Strong Gravitational Lensing

Let us comment briefly on strong gravitational lensing in the context of MDM and MOND. It is known that the critical surface density required for strong lensing is $\Sigma_{c}=\;\frac{1}{4\pi }\;\frac{cH_{0}}{G}\;F\left(z_{l},z_{s}\right)$, with F≈10 for typical clusters and background sources at cosmological distances. Sanders argued that, in the deep MOND limit, $\Sigma_{MOND}\approx a_{c}/G$ [66]. Recalling that numerically $a_{c}\approx cH_{0}/6$, Sanders concluded that MOND cannot produce strong lensing on its own: $\Sigma_{c}\approx 5\Sigma_{MOND}$. On the other hand, MDM mass distribution is expected to be sufficient for strong lensing since the natural scale for the critical acceleration for MDM is $a_{0}=cH_{0}=2\pi a_{c}\approx 6a_{c}$, five to six times that for MOND [67].

MDM as Puffy Dark Matter

As shown above, MDM quanta obey infinite statistics. Hence they are extended (see Appendix B) and the well-known tools of effective field theory are inadequate. How they interact with ordinary matter and how they self-interact remain to be investigated. On the other hand, we can heuristically argue that MDM may enjoy similar properties as DM that are known to have finite size (and hence, in a way, extended). One such type of DM is the Puffy DM [68].

Collision-less CDM predictions are known to be in tension with small scale structure observations. Self-interacting dark matter (SIDM) models [41] have been proposed to address these problems of ΛCDM; and observations seem to require DM self-scatter with a cross-section decreasing with velocity. Puffy DM naturally satisfies this observational constraint by having a finite size that is larger than its Compton wavelength. It has been shown to be successful [68] in explaining observations across a wide range of mass scales spanning dwarf galaxies of the THINGS sample, low-surface-brightness spiral galaxies and clusters of galaxies including the Bullet Cluster. It remains to be seen if MDM enjoys similar successes but the prospects look promising.

## 7. Turbulence and Spacetime Foam

In fully developed turbulence in three spatial dimensions, Kolmogorov scaling specifies the behavior of *n*-point correlation functions of the fluid velocity. The scaling [69,70] follows from the assumption of constant energy flux, $\frac{v^{2}}{t}∼\varepsilon$, where *v* stands for the velocity field of the flow, and the single length scale *ℓ* is given as ℓ∼v·t. This implies that $v∼{\left(\varepsilon \;\ell \right)}^{1/3}\;,$ consistent with the experimentally observed two-point function $\langle v^{i}\left(\ell \right)v^{j}\left(0\right)\rangle ∼{\left(\varepsilon \;\ell \right)}^{2/3}\delta^{ij}$.

In this section we will show that there are deep similarities between the problem of quantum gravity and turbulence [71]. The connection between these seemingly disparate fields is provided by the role of diffeomorphism symmetry in classical gravity and the volume preserving diffeomorphisms of classical fluid dynamics. Furthermore, in the case of irrotational fluids in three spatial dimensions, the equation for the fluctuations of the velocity potential can be written in a geometric form [72] of a harmonic Laplace–Beltrami equation: $\frac{1}{\sqrt{-g}}\partial_{a}\left(\sqrt{-g}g^{ab}\partial_{b}\varphi \right)=0\;$. Here, apart from a conformal factor, the effective space time metric has the canonical ADM form $ds^{2}=\frac{\rho_{0}}{c}\left[c^{2}dt^{2}-\delta_{ij}\left(dx^{i}-v^{i}dt\right)\left(dx^{j}-v^{j}dt\right)\right]$, where *c* is the sound velocity. We observe that in this expression for the metric, the velocity of the fluid $v^{i}$ plays the role of the shift vector $N^{i}$ which is the Lagrange multiplier for the spatial diffeomorphism constraint (the momentum constraint) in the canonical Dirac/ADM treatment of Einstein gravity: $ds^{2}=N^{2}dt^{2}-h_{ij}\left(dx^{i}+N^{i}dt\right)\left(dx^{j}+N^{j}dt\right)$. Hence in the fluid dynamics context, $N^{i}\to v^{i}$ and a fluctuation of $v^{i}$ would imply a fluctuation of the shift vector. This is possible provided the metric of spacetime fluctuates, which is a very loose, intuitive, semi-classical definition of the quantum foam.

Next recall length fluctuations $\delta \ell ∼\ell^{1/3}\ell_{P}^{2/3}$. If one defines the velocity as $v∼\frac{\delta \ell }{t_{c}}$, where the natural characteristic time scale is $t_{c}∼\frac{\ell_{P}}{c}$, then it follows that $v∼c{\left(\frac{\ell }{\ell_{P}}\right)}^{1/3}$. It is now obvious that a Kolmogorov-like scaling [69,70] in turbulence has been obtained, that is, the velocity scales as $v∼\ell^{1/3}$ and the two-point function has the needed two-thirds power law. Since the velocities play the role of the shifts, they describe how the metric fluctuates at the Planck scale. The implication is that at short distances, spacetime is a chaotic and stochastic fluid in a turbulent regime [73] with the Kolmogorov length *l*. This interpretation of the Kolmogorov scaling in the quantum gravitational setting implies that the physics of turbulence may help us understand the quantum fluctuation phase of strong quantum gravity.

## 8. Summary and Discussion

We have argued that the laws of physics that determine the precision with which the geometry of spacetime can be measured also limit the power of and the amount of information contained in black hole computers. Furthermore, the physics of spacetime fluctuations also yields a(n arguably) successful dark energy model in terms of an effective positive cosmological constant (related to the Hubble parameter). Then we show that gravitational thermodynamics/ entropic gravity arguments, generalized to a spacetime (like ours) with dark energy imitating a (positive) cosmological constant, lead to a dark matter model which relates dark energy, dark matter, and ordinary (baryonic) matter and is remarkably consistent with observations at both the galactic and cluster scales. Lastly we show that turbulence is intimately related to properties of spacetime foam in the gravitational context. These results spanning black holes, computers, space-time foam, dark energy, dark matter and turbulence are testimony to the unity of nature. They demonstrate the conceptual interconnections of fundamental physics which makes crucial (explicit or implicit) use of the concept of entropy and the dynamics of gravitation.

The confluence of entropy and gravitation has produced some rather novel results. We would argue that none is more intriguing than the manifestation that both dark energy and dark matter have their origins in quantum gravity and that their quanta obey infinite statistics while ordinary particles obey either the Fermi or Bose statistics. This may be the main difference between the dark sector and ordinary matter. Furthermore, theories of “particles” obeying infinite statistics are non-local. (See Appendix B). So it is quite conceivable that the non-locality encoded in the holographic principle, a hallmark of quantum gravity, is related to this non-locality in infinite statistics. (We note that extremal black holes, another gravitational system, also obey infinite statistics [74,75].)

We conclude this review paper with an observation (perhaps more like a speculation). As the gravitational thermodynamics and entropic gravity ideas have hinted, gravitation may ultimately be derived from thermodynamic/entropic arguments. And if we also take seriously the recent proposal that spacetime geometry/gravitation may simply be an emergent phenomenon from quantum entanglements, as implied by the conjecture ER = EPR [76], we can certainly entertain the idea that even quantum mechanics could be related to thermodynamics in a deep and unfathomable way [77,78]. If so, then it follows that thermodynamics, Einstein’s “meta-theory”, may hold the key to formulating as well as understanding the ultimate physical laws; and reigning supreme will be its protagonist—entropy.

## Appendix A. Energy-Momentum Fluctuations and Possible Tests of Spacetime Foam

### Appendix A.1. Energy-Momentum Fluctuations

Just as there are uncertainties in spacetime measurements, there are also uncertainties in energy-momentum measurements due to spacetime foam effects [5,6]. Imagine sending a particle of momentum *p* to probe a certain structure of spatial extent *l* so that p∼ℏ/l. It follows that $\delta p∼(\hbar /l^{2})\delta l$. Spacetime fluctuations $\delta l≳l{\left(l_{P}/l\right)}^{2/3}$ can now be used to give $\delta p≳p{\left(\frac{p}{m_{P}c}\right)}^{2/3}$ and $\delta E≳E{\left(\frac{E}{E_{P}}\right)}^{2/3}$, where $E_{P}=m_{P}c^{2}∼10^{19}$ GeV is the Planck energy. Consequently the *dispersion relation* is now modified [26] to read $E^{2}-p^{2}c^{2}-\epsilon p^{2}c^{2}{\left(\frac{pc}{E_{P}}\right)}^{2/3}=m^{2}c^{4}$, for high energies with $E\gg mc^{2}$, with ϵ∼1. This modified dispersion relation, in turn, leads to a *fluctuating speed of light* [26,79]: $v=\frac{\partial E}{\partial p}≃c\left(1+\frac{5}{6}\epsilon \frac{E^{2/3}}{E_{P}^{2/3}}\right)$, which is energy-dependent and fluctuates around c.

### Appendix A.2. Possible Ways to Test Spacetime Foam

There have been numerous proposals to detect spacetime foam, involving astronomical high-energy gamma ray observations of distant gamma-ray bursts and distant quasars, gravity-wave interferometers and atom interferometers and so forth. But when the proper averaging is carried out (even if there is such a formalism), now it appears (at least to this author) that the fluctuations are perhaps too small to be detectable with the currently available experimental and observational techniques. Nevertheless, let us briefly discuss several of the proposals to detect spacetime foam.

I. Observing gamma rays from extragalactic sources:

For photons emitted simultaneously from a distant source, we expect an energy-dependent spread in their arrival times. So one idea is to look for a noticeable spread in arrival times for high energy gamma rays from distant gamma ray bursts (GRB). This proposal was first made by G. Amelino-Camelia et al. [79] in another context. But the time-of-flight differences δt increase only with the cube root of the average overall time *t* of travel ($\delta t∼t^{1/3}t_{P}^{2/3}$) from the gamma ray bursts to our detector, leading to a time spread too small to be detectable [26].

Another way is to find out if spacetime foam-induced phase incoherence of light from a distant galaxy or GRB can make the light wave front noticeably distorted so as to lose the sharp ring-like interference pattern around the galaxy or GRB; [80,81] or to look for halo structures in the interferometric fringes induced by fluctuations in the directions of the wave vector of light from extragalactic sources [82].

More recently, my collaborators and I [83] showed explicitly how wavefront distortions on small scales cause the image intensity to decay to the point where distant objects become undetectable when the path-length fluctuations become comparable to the wavelength of the radiation. We noted that detections of quasars at TeV energies with ground-based Cherenkov telescopes seem to have ruled out the holographic spacetime foam model (with δl scaling as $l^{1/3}l_{P}^{2/3}$). But this claim is subject to some caveats. For example, my collaborators and I considered only the instantaneous fluctuations in the distance between the location of the emission and a given point on the telescope aperture. Perhaps one should average over both the huge number of Planck timescales during the time it takes light to propagate through the telescope system and over the equally large number of Planck squares across the detector aperture. It is then possible that the net fluctuations are exceedingly small; but at the moment, to the best of my knowledge, there is no formalism for carrying out such averages [84].

II. Measuring the foaminess of spacetime with laser-based interferometers:

For an interferometer with bandwidth centered at frequency *f*, the relevant length scale characteristic of the noise due to space-time foam is given by $l_{P}^{2/3}{\left(c/f\right)}^{1/3}$. This uncertainty manifests itself as a displacement noise (in addition to noises from other sources) that infests the interferometers. The hope is that modern gravitational-wave interferometers, through future refinements, may reach displacement noise level low enough to test a subset of the space-time foam models [85,86,87]. But this hope is based on the assumption that spacetime in between the mirrors in the interferometer fluctuates coherently for all the photons in the beam. However the large beam size in LIGO (compared to the Planck scale) makes such coherence unlikely.

## Appendix B. Infinite Statistics

For completeness, here we list some of the properties of infinite statistics [27,28,29]. A Fock realization of infinite statistics is given by $a_{k}a_{l}^{†}=\delta_{k,l}$. This algebra, known as Cuntz algebra, is described by the average of the bosonic and fermionic algebras. Any two states obtained by acting on |0> with creation operators in different order are orthogonal to each other: $<0|a_{i1}...a_{iN}a_{jN}^{†}...a_{j1}^{†}|0>=\delta_{i1,j1}...\delta_{iN,jN}$, implying that particles obeying infinite statistics are distinguishable. Accordingly, the partition function is given by $Z=\Sigma e^{-\beta H}$, without the Gibbs factor. It is known that, in infinite statistics, all representations of the particle permutation group can occur. Theories of particles obeying infinite statistics are non-local [29,88]. (To be more precise, the fields associated with infinite statistics are not local, neither in the sense that their observables commute at spacelike separation nor in the sense that their observables are pointlike functionals of the fields.) In fact, the number operator $n_{i}$ (which, we recall, satisfies the condition $n_{i}a_{j}-a_{j}n_{i}=-\delta_{i,j}a_{j}$)

(A1) $$n_{i}=a_{i}^{†}a_{i}+\sum_{k}a_{k}^{†}a_{i}^{†}a_{i}a_{k}+\sum_{l}\sum_{k}a_{l}^{†}a_{k}^{†}a_{i}^{†}a_{i}a_{k}a_{l}+...,$$

and Hamiltonian and so forth, are both nonlocal and nonpolynomial in the field operators. It is also known that TCP theorem and cluster decomposition still hold; and quantum field theories with infinite statistics remain unitary [29].
